# GREATER ADHERENCE TO DAILY HEALTH BEHAVIORS RELATE TO BETTER COGNITIVE FUNCTION

**DOI:** 10.1093/geroni/igad104.0925

**Published:** 2023-12-21

**Authors:** Fumiko Hamada, Soomi Lee

**Affiliations:** University of South Florida, Tampa, Florida, United States; The Pennsylvania State University, University Park, Pennsylvania, United States

## Abstract

Healthy life habits cannot be formed overnight; therefore, adherence to optimal sleep, physical activity, and healthy eating behaviors is vital. This paper examined the relationship between the adherence rate of the three key health behaviors (i.e., sleep, physical activity, and healthy diet) and cognitive function in adulthood. Data were retrieved from Midlife in the United States third wave (MIDUS 3) Project 2, National Study of Daily Experiences (n=1,061). For eight consecutive days, participants reported nightly sleep duration, daily amount of physical activity, and daily consumption of fast food. Adherence rates of health behaviors were calculated by the sum of having optimal sleep (6 < sleep hours ≤ 9), any intensity of physical activity (60 min.≤), and not eating fast food (yes/no) across the study period. Participants also completed a Brief Test of Adult Cognition by Telephone (BTACT). A series of general linear models examined the associations of the three adherence variables with the overall BTACT cognition score adjusting for sociodemographic and health covariates. Greater adherence rates of optimal sleep (𝛽=0.02; SE=0.01; p=0.01), physical activity (𝛽=0.03; SE=0.01; p<0.01 ), and not eating fast food (𝛽=0.02; SE=0.01; p=0.02) were each and associated with higher BTACT score. Among the three adherence variables, adherence to physical activity was the most important for cognition independent of other health behaviors. Consistent associations were found for executive function, but not episodic memory. These findings suggest that adults with greater adherence to a healthy lifestyle have better cognitive function, and consistency of healthy behaviors is vital for cognitive health.

